# Pharmacist participation as providers in Ohio Medicaid: an analysis of claims, services, and billing patterns, 2021-2025

**DOI:** 10.1093/haschl/qxag126

**Published:** 2026-05-21

**Authors:** E Michael Murphy, Stuart J Beatty, Macarius M Donneyong, Henry J Mann

**Affiliations:** Division of Pharmacy Practice and Science, The Ohio State University College of Pharmacy, Columbus, OH 43210, United States; Government Affairs, American Pharmacists Association, Washington, DC 20037, United States; Raabe College of Pharmacy, Ohio Northern University, Ada, OH 45810, United States; Division of Pharmacy Practice and Science, The Ohio State University College of Pharmacy, Columbus, OH 43210, United States; Division of Pharmacy Practice and Science, The Ohio State University College of Pharmacy, Columbus, OH 43210, United States

**Keywords:** Medicaid, pharmacist, pharmacy, primary care, workforce, claims analysis

## Abstract

**Introduction:**

States increasingly recognize pharmacists as medical-benefit providers, yet participation under Medicaid is not well documented.

**Methods:**

We conducted a retrospective descriptive analysis of pharmacist-submitted claims to the Ohio Department of Medicaid from January 2017 to January 2025. Claims included current procedural terminology/International Classification of Diseases-10 codes, paid/denied status, payment amounts, and place of service.

**Results:**

Pharmacists submitted 1424 claims before implementation and nearly 250 000 afterward, with more than 85% paid and $4.4 million reimbursed. Most claims occurred in team-based settings (Federally Qualified Health Centers [55%] and office practices [41%]) while community pharmacy billing was rare (0.19%). Common services included evaluation and management visits, preventive counseling, and continuous glucose monitoring; leading diagnoses were type 2 diabetes, hypertension, and nicotine dependence.

**Conclusion:**

Recognition of pharmacists as medical-benefit providers produced rapid participation at modest cost, primarily in integrated clinical settings. These findings suggest that streamlined referral processes and improved billing support may expand participation, particularly in community pharmacies.

## Introduction

Pharmacists' roles in the United States have expanded over past decades, evolving from a focus on product dispensing to the provision of direct patient care services. Since the 1990s, state legislatures have broadened pharmacists' scope of practice to align legal authority with clinical training. Despite this clinical expansion, pharmacists have historically not been recognized as reimbursable medical-benefit providers by most insurers.^1,2^

Despite decades of advocacy,^3,4^ and policy recommendations from the US Surgeon General's Office and the National Governors Association,^5,6^ Medicare Part B has not adopted direct reimbursement for pharmacists' services. However, over the past 2 decades, state Medicaid programs and commercial payers began recognizing pharmacists as medical-benefit providers and reimbursing clinical services. Early initiatives in states such as Virginia,^7^ Minnesota,^8^ and Washington^9,10^ demonstrated the feasibility of pharmacist medical benefit reimbursement by Medicaid and commercial plans. In 2021, the Ohio Department of Medicaid (ODM) enacted the ability for pharmacists to be reimbursed, a contemporary shift, that led to the adoption of comparable programs in other states over subsequent years.^11^

Under Ohio Medicaid,^12^ after a referral is received, pharmacists may bill for services under a collaborative practice agreement, or for immunization/medication administration. Payment is made inline with other Medicaid providers in the state and until recently, no payment was made for pharmacist services delivered in certain health system settings.^13^

Despite this policy momentum, little is known about the extent to which pharmacists participate in programs. Prior initiatives, including medication therapy management pilot programs and value-based care arrangements, have examined pharmacist participation and implementation within specific program designs using small samples and mixed-methods approaches; however, these studies were not designed to evaluate claims-level billing, payment, and denial patterns across a statewide population of pharmacists.^14-16^ Wittenauer et al.^17^ found that pharmacist-submitted medical-benefit vaccination claims were exceedingly rare, even in states with laws that mandate commercial payers to enroll and pay pharmacists as medical-benefit providers, indicating that pharmacists are not routinely billing for these services despite having legal authority to do so. This evidence points to a vital implementation gap. Yet no published research has examined pharmacist participation in Medicaid medical-benefit billing programs, nor has any study characterized the types of services provided, patterns of claim submission and denial, or associated payment amounts beyond vaccination-specific contexts.

This study addresses these gaps by analyzing Ohio pharmacist-submitted Medicaid claims from January 2017 through January 2025. We quantified the extent of pharmacist participation, identified service categories, assessed payment and denial patterns, and examined variations across care settings. Understanding these patterns is essential for informing Medicaid program design, reimbursement policies, and workforce planning as pharmacists become increasingly integrated into primary care and chronic disease management.

## Methods

This study is a retrospective descriptive analysis of pharmacist-submitted claims to ODM spanning January 17, 2017, through January 17, 2025. Inferential trend testing was intentionally not performed. The objectives of this study were to 1) assess the number of pharmacists that have submitted claims as Medicaid providers in Ohio, (2) analyze the frequency and types of International Classification of Diseases (ICD) codes and current procedural terminology (CPT) codes billed by pharmacists, (3) determine the total reimbursement of pharmacist-provided services by ODM, and (4) evaluate trends in pharmacist billing over time. To accomplish this, a comprehensive dataset was obtained from ODM that captured all pharmacist-rendered services across both fee-for-service and managed care. The dataset included counts of paid/denied claims, procedure and diagnosis codes, payment amounts, and place-of-service (POS) designations. Data were requested for an equivalent period before and after program implementation on January 17, 2021, to permit longitudinal comparison. Before this date, pharmacists were not recognized as medical-benefit providers under Ohio Medicaid; therefore, any claims submitted before 2021 represent isolated circumstances (eg, pilot programs) rather than routine participation.

ODM constructed the analytic dataset by identifying all pharmacists enrolled as providers and extracting all claims submitted by those individuals under both fee-for-service and managed care plans. A deidentified unique provider identifier for each pharmacist was created and linked to all associated claims of that identifier. To comply with privacy regulations,^18-21^ ODM removed all geographic detail, suppressed small cell sizes, and provided data in yearly time blocks. Because this dataset excludes rare services or low-volume providers, it represents a conservative estimate of the extent of pharmacist participation. Additionally, because pharmacists are required to obtain a referral before providing services, some services rendered in practice may not result in a billable encounter if referral pathways are inconsistent or poorly integrated.

Before analysis, all claims underwent a structured data-quality review, including assessment for completeness, internal consistency, duplicate records, invalid or missing fields, and anomalies in adjudication amounts. ICD, CPT, and POS code fields were standardized to ensure uniform interpretation across settings. Definitions for CPT codes were verified using the American Medical Association's CPT Handbook,^22^ and POS codes were cross-walked to the Centers for Medicare & Medicaid Services (CMS) POS code set to align service-location classification with national standards.^23^ Payment fields were normalized for comparability, and outlier checks were performed to identify implausible values.

To characterize the extent and nature of pharmacist engagement with Medicaid over time, we used basic descriptive summaries (frequency and percent) to assess the patterns and trends of pharmacist-submitted claim volumes, service mix, and payment and denial patterns. Service patterns were compared across POS, including, but not limited to, Federally Qualified Health Centers (FQHCs), office-based practices, pharmacies, telehealth encounters, and home-based care. Trends across the pre- and postintervention periods were examined to identify changes in pharmacist participation following program implementation.

In interpreting these data, it is essential to note that pharmacists could submit claims not only for clinical services but also for certain products, including vaccines, laboratory tests, and select medications. These product claims typically occurred when a pharmacist-delivered service involved the administration or provision of an associated item. Pharmacists also submitted CPT Category II (CPT II) codes, which are optional alphanumeric tracking codes used to document quality or performance metrics and are usually not tied to direct reimbursement. Because the dataset includes service claims, product claims, and nonreimbursable CPT II codes, total claim volume should not be interpreted as a one-to-one measure of reimbursable pharmacist services.

## Results

Before the statewide pharmacist medical benefit program began on January 17, 2021, pharmacist participation in Medicaid billing was minimal. During the preintervention period (January 17, 2017 to January 16, 2021), pharmacists submitted only 1424 claims, 72% of which occurred in the final year before program implementation. These preprogram claims represent atypical or pilot-driven billing rather than routine pharmacist participation and provide important context for interpreting the substantial increase in claims submitted after 2021.

Following implementation in 2021, pharmacist engagement expanded rapidly. The 4-year period examined in this study reveals a clear and growing role for pharmacists. Between 2021 and 2025, pharmacists submitted nearly a quarter-million claims with over 85% ultimately paid. This resulted in $4.4 million in reimbursements, with $1.7 million in denied payments. Some pharmacists billed infrequently, submitting only a handful of claims over multiple years. Others, however, emerged as high-volume clinical contributors, with individual totals exceeding 20 000 claims and generating several hundred thousand dollars in Medicaid reimbursement. Only 268 (1.96%) pharmacists of the estimated 13 700 employed pharmacists in Ohio^24^ submitted at least one claim during the study period.

Where pharmacists delivered services proved highly consequential. Table 1 shows that FQHCs accounted for 55.34% of claims and offices accounted for 41.35% of claims, with telehealth, home, pharmacy, and other sites comprising the remainder. FQHCs and office settings not only drove claim volume but also accounted for most of the payments pharmacists received. Claims submitted at community pharmacies, the location where the highest number of pharmacists' practice,^25^ accounted for only 0.19% of overall claims during the study period, with little year-over-year growth.

**Table 1:** Submitted pharmacist claims by place of service January 17, 2021 to January 17, 2025.

Place of service	Total claims submitted	Claims paid, *n* (%)^a^	Claims denied, *n* (%)^a^	Payment made ($)	Payment denied ($)
FQHC	135 892	117 778 (87)	18 114 (13)	2 498 217	857 779
Office	101 548	86 329 (85)	15 219 (15)	1 758 610	711 099
Home	1206	1009 (84)	197 (16)	58 077	20 431
TPOPH	3231	1814 (56)	1417 (44)	44 620	63 716
MIC	164	146 (89)	18 (11)	8141	1275
OPS	282	246 (87)	36 (13)	7297	1930
Pharmacy	460	368 (80)	92 (20)	7076	3755
Other^b^	2793	1665 (60)	1128 (40)	14 123	54 154
**Total**	245 576	209 355 (85)	36 221 (15)	4 396 161	1 714 139

Abbreviations: FQHC, federally qualified health center; TPOPH, Telehealth Provided Other than in Patients Home; MIC, Mass Immunization Center; OPS, Other Place of Service.

^a^Percent calculated from total claims submitted in place of service category.

^b^Other includes the following places of service: Group Home, Independent Laboratory, Indian Health Service Free-standing Facility, Indian Health Service Provider-based Facility, Mobile Unit, Nursing Facility, Off Campus-Outpatient Hospital, On Campus-Outpatient Hospital, Residential Substance Abuse Treatment Facility, Skilled Nursing Facility, and Telehealth Provided in Patient's Home.

The distribution of billed services highlights 3 policy-relevant patterns (Table 2). First, evaluation and management (E/M) office or other outpatient services dominated pharmacist billing, with CPT codes 99212 and 99213 accounting for the largest share of submitted claims and payments, indicating pharmacists' primary role in routine outpatient chronic care management. Second, preventive counseling services, particularly CPT 99401, comprised a substantial share of paid claims with very high payment rates, reflecting pharmacists' involvement in lifestyle modification and risk-factor management, including tobacco cessation and chronic disease self-management. Third, in FQHCs, the all-inclusive encounter code T1015 accounted for a disproportionate share of both claim volume and reimbursement, reinforcing the central role of encounter-based payment in facilitating pharmacist participation in team-based settings. Other billed services, including telephone E/M codes, immunization administration, continuous glucose monitoring, and injectable therapies, occurred at much lower volumes and are detailed in Table 2.

**Table 2:** Most common submitted procedure codes by payment made January 17, 2021 to January 17, 2025.

Description	Total claims submitted	Claims paid, *n* (%)	Claims denied, *n* (%)	Payment made ($)	Payment denied ($)
Office or other outpatient services					
Office or other outpatient visit, straightforward level of medical decision-making, 15-29 minutes (CPT: 99202)	736	484 (66)	252 (34)	13 962	12 420
Office or other outpatient visit, low level of medical decision-making, 30-44 minutes (CPT: 99203)	741	488 (66)	253 (34)	19 576	18 936
Office or other outpatient visit, <10 minutes (CPT: 99211)	5061	4057 (80)	1004 (20)	29 864	10 783
Office or other outpatient visit, straightforward level of medical decision-making, 10-19 minutes (CPT: 99212)	25 022	19 918 (80)	5104 (20)	290 936	131 900
Office or other outpatient visit, low level of medical decision-making, 20-29 minutes (CPT: 99213)	44 884	38 170 (85)	6714 (15)	878 836	394 290
Office or other outpatient visit, moderate level of medical decision-making, 30-39 minutes (CPT: 99214)	977	573 (59)	404 (41)	19 770	42 311
**Counseling risk factor reduction and behavior change intervention**					
Preventative medicine counseling, 15 minutes (CPT: 99401)	8879	8810 (99)	69 (1%)	351 500	2582
Preventative medicine counseling, 30 minutes (CPT: 99402)	641	634 (99)	7 (1)	31 760	380
Preventative medicine counseling, 45 minutes (CPT: 99403)	171	164 (96)	7 (4)	10 226	656
Preventative medicine counseling, 60 minutes (CPT: 99404)	178	175 (98)	3 (2)	11 296	160
**Telephone services**					
Telephone evaluation and management service, 5-10 minutes (CPT: 99441)	3921	3688 (94)	233 (6)	26 422	5686
Telephone evaluation and management service, 11-20 minutes (CPT: 99442)	5598	5027 (90)	571 (10)	77 301	19 667
**Immunization administration for vaccines/toxoids**					
Immunization administration (CPT: 90471)	1000	853 (85)	147 (15)	7256	4330
Immunization administration of COVID-19 vaccine, first dose (CPT: 0001A)	156	124 (79)	32 (21)	10 875	3200
Immunization administration of COVID-19 vaccine, first dose (CPT: 0011A)	293	279 (95)	14 (5)	14 141	1250
Immunization administration of COVID-19 vaccine, second dose (CPT: 0012A)	262	249 (95)	13 (5)	11 359	1180
Immunization administration of COVID-19 vaccine (CPT: 0071A)	77	76 (99)	1 (1)	6800	100
**Endocrinology**					
Ambulatory continuous glucose monitoring, provided equipment sensor placement (CPT: 95250)	489	368 (75)	121 (25)	10 482	2051
Ambulatory continuous glucose monitoring analysis, interpretation and report (CPT: 95251)	1242	1094 (88)	148 (12)	7692	4175
**Therapeutic, prophylactic, and diagnostic injections and infusions**					
Therapeutic, prophylactic, or diagnostic injection (CPT: 96372)	959	747 (78)	212 (22)	7812	7974
**Temporary national codes established by Medicaid**					
Clinic visit/encounter, all-inclusive (CPT: T1015)	25 171	19 224 (76)	5947 (24)	2 399 528	857 359

Abbreviation: CPT, current procedural terminology.

Diagnosis patterns aligned closely with the services delivered. Table 3 illustrates that type 2 diabetes mellitus emerged as the single most common diagnosis associated with pharmacist claims, followed by hypertension and nicotine dependence. Encounters tied to immunizations, substance use disorders, and other preventative indications also appeared frequently. Across most POS categories, the same core diagnostic patterns prevailed, indicating consistent pharmacists' clinical focus.

**Table 3:** Most commonly submitted diagnosis codes by payment made January 17, 2021 to January 17, 2025.

Description	Total claims submitted	Claims paid, *n* (%)	Claims denied, *n* (%)	Payment made ($)	Payment denied ($)
Type 2 diabetes mellitus (ICD: E11)	138 379	118 971 (86)	19 408 (14)	2 259 416	844 124
Encounter for other special exam w/o complaint, suspected or reported dx (ICD: Z01)	19 080	18 875 (99)	205 (1)	405 796	3956
Essential (primary) hypertension (ICD: I10)	15 582	13 042 (84)	2540 (16)	262 441	112 852
Nicotine dependence (ICD: F17)	8058	6398 (79)	1660 (21)	165 036	97 135
Type 1 diabetes mellitus (ICD: E10)	6861	5955 (87)	906 (13)	117 018	42 762
Unspecified viral hepatitis (ICD: B19)	2491	2092 (84)	399 (16)	93 503	29 315
Encounter for immunization (ICD: Z23)	2842	2359 (83)	483 (17)	92 139	37 576
Problems related to lifestyle (ICD: Z72)	5771	4950 (86)	821 (14)	83 797	27 914
Long term (current) drug therapy (ICD: Z79)	4612	3808 (83)	804 (17)	81 659	36 075
Chronic viral hepatitis (ICD: B18)	2755	2328 (85)	427 (15)	77 730	29 292
Opioid related disorders (ICD: F11)	3992	2708 (68)	1284 (32)	62 147	46 831
Overweight and obesity (ICD: E66)	1587	1187 (75)	400 (25)	43 075	32 574
HIV disease (ICD: B20)	1772	1384 (78)	388 (22)	41 416	30 296
Elevated blood glucose level (ICD: R73)	2169	1830 (84)	339 (16)	36 710	14 092
Alcohol-related disorders (ICD: F10)	1738	1122 (65)	616 (35)	31 180	21 894
Persons encountering health services for other counseling and medical advice not elsewhere classified (NEC) (ICD: Z71)	1856	1328 (72)	528 (28)	28 521	22 435
Encounter other prophylactic measures (ICD: Z29)	1495	1264 (85)	231 (15)	28 335	18 504
Other anxiety disorders (ICD: F41)	977	765 (78)	212 (22)	22 782	15 449
Schizoaffective disorders (ICD: F25)	391	270 (69)	121 (31)	22 227	6354
Presence of cardiac and vascular implants and grafts (ICD: Z95)	1143	1043 (91)	100 (9)	20 702	3636

Abbreviations: ICD, International Classification of Diseases; HIV, human immunodeficiency virus.

**Figure 1:**
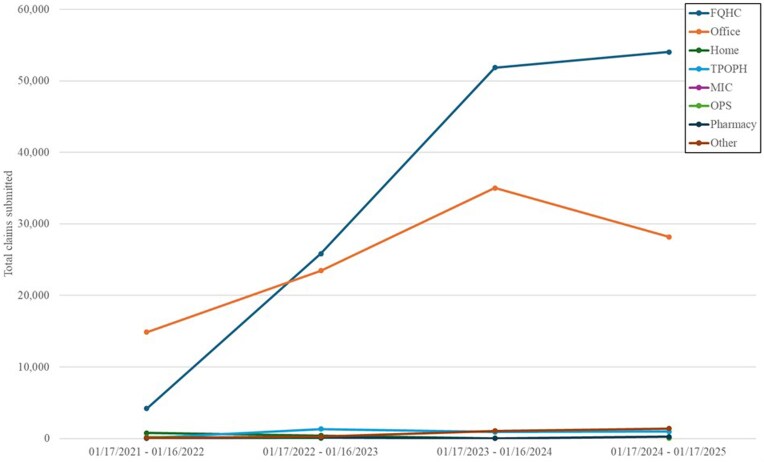
Total pharmacist claims submitted by place of service over time periods. Federally Qualified Health Center (FQHC); Telehealth Provided Other than in Patients Home (TPOPH); Mass Immunization Center (MIC); Other Place of Service (OPS). Other includes the following places of service: Group Home, Independent Laboratory, Indian Health Service Free-standing Facility, Indian Health Service Provider-based Facility, Mobile Unit, Nursing Facility, Off Campus-Outpatient Hospital, On Campus-Outpatient Hospital, Residential Substance Abuse Treatment Facility, Skilled Nursing Facility, and Telehealth Provided in Patient's Home.

Trends over the 4 years are described in Figure 1 and show rapid growth in the number of pharmacist-submitted claims, particularly within FQHCs, where annual claims rose from just over 4000 early in the study period to more than 54 000 by 2024-2025. Office-based practice claims also grew substantially before leveling off slightly in the most recent year. Telehealth claims spiked in 2022-2023, reflecting a period of heightened remote care delivery before stabilizing at moderate levels. These trends indicate that the pharmacist workforce is increasingly integrated into Medicaid care environments, contributing meaningfully to chronic disease management, preventive counseling, and team-based primary care.

## Discussion

While there has been a recent surge of policy changes in multiple states^10^ to allow pharmacist reimbursement for services, it remains unclear how many pharmacists have engaged in these new programs and how program design may influence participation. In this retrospective descriptive analysis in Ohio, nearly 250 000 pharmacist claims were submitted, resulting in a cost to the state of $4.4 million over 4 years. Claims submitted by pharmacists prior to 2021 were rare, likely reflecting activity associated with ODM managed care pilot initiatives.^26^ Following program implementation, the claims started small, at 20 328 but increased to 85 855 by the final year of the study period. One reason often cited for limiting the inclusion of pharmacists in provider networks is the potential financial burden of all practicing pharmacists submitting claims following law changes. The Ohio experience suggests a more measured pattern of uptake, with participation concentrated among a relatively small subset of pharmacists practicing in offices and FQHCs, while community pharmacy settings billed fewer than 500 total claims over 4 years. One particular office setting in Ohio analyzed internal data following the implementation of the program and found that billable encounters by pharmacists increased gradually from 1863 to 7590 over the first 3 years.^27^

The low level of community pharmacist participation may be attributed to a set of interrelated policy barriers rather than a lack of pharmacist capacity or patient need. Previous analysis in Ohio showed that barriers to pharmacist implementation did exist, and specifically named system integration and patient eligibility as problems in this setting.^28^ In Ohio, pharmacists must obtain referrals and operate under collaborative practice agreements, requirements that are comparatively easier to meet in office-based practices and FQHCs where pharmacists share patient panels, electronic health records (EHRs), and established workflows with a limited number of prescribers. In contrast, community pharmacists typically interact episodically with patients, lack direct EHR access, and must coordinate referrals across multiple unaffiliated prescribers, conditions that complicate patient identification, documentation, and claim submission. Together, referral complexity, collaborative practice agreement (CPA) requirements, EHR disconnection, and workflow misalignment create cumulative friction that may explain limited community pharmacy-based billing despite community pharmacists' broad accessibility.

These implementation dynamics may help explain why pharmacist participation scaled primarily within team-based care environments rather than community pharmacies, even as policymakers increasingly view pharmacists as a means of expanding access amid primary care shortages. They also suggest that payment for pharmacists' services alone may be insufficient to enable broad participation without concurrent attention to workflow integration, data connectivity, and referral design—particularly in community pharmacy settings that serve rural and underserved populations. States and payers with less restrictive policies, different referral pathways, or stronger data integration may observe different participation patterns, warranting the need for comparative evaluations.

In this study, the most common diagnoses involved patients with type 2 diabetes mellitus, essential hypertension, and nicotine dependence. There is significant spending on these chronic diseases,^29,30^ for example the annual cost of diabetes in 2022 was $412.9B.^31^ Agencies have called for a multidisciplinary approach to treat these and other chronic diseases to improve health outcomes and decrease costs. One estimate indicates pharmacists could save the US healthcare system more than $1T over 30 years if better utilized.^32^ Pharmacists in Ohio appear to be incentivized to provide care in these team-based settings for those with chronic disease, possibly replacing visits with other healthcare professionals. Evidence from other team-based care models supports this substitution hypothesis reduce total costs, lower acute-care utilization, and improve quality, rather than increasing overall spending.^33^ Future studies should evaluate pharmacists ability to absorb routine chronic care workload of other providers similarly without significantly increasing overall costs. Causal substitution cannot be inferred from claims alone and warrants further evaluation through claims analysis of pharmacists and other healthcare professionals.

Pharmacists in this study were most likely to utilize CPT codes 99212 and 99213. These codes align well with the chronic disease management ICD codes that were submitted in the practice settings. Unlike other states, Ohio limited pharmacists to bill claims above 99213 outside of rare situations.^34,35^ Where states allow higher-level E/M billing for pharmacists, policymakers should ensure that coding guidance aligns with clinical complexity to avoid unintentionally capping pharmacists' contributions. Clarifying coding scope and documentation standards (with payer education and billing support) may accelerate appropriate use without encouraging upcoding.

Similar to results found by Wittenauer et al.,^17^ this study did see pharmacists utilizing medication and immunization codes for patients, albeit at a lesser rate than those for E/M. Separate pathways exist for reimbursement of immunizations under the pharmacy benefit, with pharmacists already equipped and integrated into workflows to submit claims, which likely explains the low number of such claims submitted. Streamlining reimbursement pathways to ensure consistency across all provider groups for medication administration will be critical. As more medications require monitoring and administration by a healthcare professional, it will be important that pharmacists be incentivized to provide this care given their accessibility to patients.

This study has several limitations. First, the analysis is a retrospective descriptive claims study and cannot be used to infer causality. Observed trends in participation, service mix, and payment patterns should therefore be interpreted as hypothesis-generating rather than causal. Second, the analysis relies on administrative claims data, which are generated for billing rather than research purposes and thus are subject to miscoding, omissions, and variable completeness. Third, the claims system does not allow authors to determine with certainty whether denied payment amounts correspond exclusively to distinct denied claims; consequently, some encounters may appear in both the paid and denied categories, complicating interpretation of denial patterns. Fourth, ODM removed data with very small counts before data release, making study estimates likely excluding a subset of low-volume activity. Fifth, the dataset captures only encounters billed through standard medical-benefit pathways; services reimbursed via alternative mechanisms, such as capitated arrangements, per-member-per-month payments, “incident-to” billing, direct patient payments, grant-funded models, or health system internal subsidies, are not comprehensively observable, likely leading to conservative estimates of service volume and reimbursement. Sixth, service-level detail in some settings (particularly FQHCs using encounter codes such as T1015) may obscure the specific mix of services delivered, and payer-specific editing rules may differentially affect observable claim submission and denial behavior. Finally, the study period (2021-2025) overlaps with the COVID-19 pandemic and its aftermath, during which evolving public-health guidance, temporary flexibilities, workforce disruptions, and shifting patient care-seeking behaviors could have influenced both the delivery of pharmacist services and billing practices in ways we cannot fully measure.

## Conclusion

Ohio's experience shows that recognizing pharmacists as Medicaid medical-benefit providers can translate into rapid, measurable participation at modest cost, concentrated in team-based clinical settings and focused on high-burden chronic conditions through ambulatory E/M, preventive counseling, and diabetes technology management. At the same time, community pharmacy billing remained rare, underscoring operational frictions, such as referral and CPA requirements, limited EHR connectivity, and payer workflow alignment, that appear easier to overcome inside integrated clinics than in pharmacy environments. For Medicaid programs, near-term policy levers include streamlining referral pathways, enhancing service-level observability under encounter payment, and providing billing/documentation support to expand participation outside clinics. Finally, these patterns are consistent with a substitution hypothesis that pharmacists absorb routine chronic care workload in team-based settings, and causal substitution cannot be inferred from claims alone and should be tested prospectively using system-level metrics.

## Supplementary Material

qxag126_Supplementary_Data
